# A Novel Approach for the Enumeration of Peripheral Blood Stem Cells Suitable for Transplantation

**DOI:** 10.1155/2014/473503

**Published:** 2014-08-05

**Authors:** Winston Costa Pereira, Omar Alsuhaibani, Ghaleb Elyamany, Abdulaziz Al Abdulaaly

**Affiliations:** Stem Cell Section, Department of CML and Blood Bank, Prince Sultan Military Medical City, P.O. Box 7897, Riyadh 11159, Saudi Arabia

## Abstract

Stem cells have the capability to proliferate and differentiate into various cells of the body. Few stem cell sources have been approved for transplantation, among them are the hematopoietic progenitor cells which are progenitors of the myeloid and erythroid lineage in the hematopoietic system, that continually provides mature blood cells during the lifespan of the individual. These well-characterized stem cells are clinically relevant in the treatment of diseases such as breast cancer, leukemias, and congenital immunodeficiencies. Peripheral blood stem transplantation is a standard procedure after its first successful transplantation more than 35 years ago. The minimum intended dose of stem cells given to the patient is 2.5 × 10^6^ –5 × 10^6^ cells. In this study, we are establishing a correlation between the number of stem cells enumerated and the weight of the patient as a determinant for suitable transplantation. We have established a conversion factor to deliver the required dose of approximately 3 × 10^6^ stem cells/kg body weight. This will ensure a uniform collection strategy that is sufficient for transplantation irrespective of the weight of the patient. This approach, if incorporated, will lead to a significantly lesser rate of bone marrow transplantation failures as sufficient number of stem cells will ensure engraftment of stem cells.

## 1. Introduction

Peripheral blood-derived stem cells (PBSCs) have been used in bone marrow transplantation ever since its first report was published in the late ‘70s [1]. In recent years, there has been rapid expansion of the clinical use of hematopoietic stem cells as well as its concomitant understanding of its basic biology. These stem cells, which are a critical component of transplantation, are progenitors to the blood cells of the body that constitutes the myeloid and erythroid lineage [2]. They continuously provide mature blood cells during the lifespan of the individual. These are one of the best characterized stem cells in the body that are clinically applicable in the treatment of diseases such as breast cancer, leukemias, and congenital immunodeficiencies [3].

Hematopoietic stem cells (HSCs) belong to a group of multipotent precursors that have a self-renewal capacity and the ability to generate different cell types that comprise of the blood-forming system [4]. Transplantation of HSCs forms the basis of consolidation therapy in cancer treatments and is used to cure or ameliorate a number of hematologic and genetic disorders [5]. HSCs are also an attractive target cell population for gene therapies because they are readily accessible for ex vivo genetic modification and allow for the possibility of sustained transgene expression in circulating peripheral blood cells throughout the lifetime of an individual [6].

PBSC transplantation (PBSCT) has become increasingly common with PBSCs largely replacing bone marrow (BM) as the preferred stem cell source due largely to quicker engraftment kinetics and ease of collection. In the peripheral blood, stem cells are found in limited numbers (less than 0.1% of all nucleated cells). Stem cell progenitor cells circulate in the periphery, as this ensures an even distribution of hematopoiesis within the bone marrow.

### 1.1. Hematopoietic Stem Cell Morphology

PBSCs consist of a subpopulation of hematopoietic progenitor cells (CD34^+^), which is morphologically difficult to identify. These cells can be distinguished by their immunophenotypic patterns as CD34^+^/CD38^−^. They do not express a full complement of either myeloid or lymphoid lineage-specific markers (Lin^−^) but do express the Thy-1 differentiation antigen. The CD34^+^/CD38^−^/Lin^−^/Thy-1^+^ cells are responsible for initiating long-term culture initiating colony (LTC-IC) assays [7].

There are many methods for stem cell quantification after collection but the most common method used today is the flow cytometric evaluation of CD34^+^ cell numbers. Enumeration of CD34^+^/CD38^−^, CD34^+^/CD33^−^, and CD34^+^/Thy-1^+^ cell subsets has proven to be a useful technique in the estimation of stem cell numbers [8]. Other methods such as colony forming units (CFU) of granulocyte-macrophage were also used to estimate stem cell numbers. This method is much less reliable due to the variation in culture techniques, media preparation, and several human factors [9].

### 1.2. Mobilization and Collection of PBSCs

Hematopoietic stem cells have an inherent property to constantly leave the bone marrow and penetrate tissues thereafter returning to the BM or peripheral niches via the blood or lymphatic system [10]. A niche is a subgroup of tissue cells and extracellular substrates that can indefinitely harbor one or more stem cells and control their self-renewal and progeny in vivo [11].

Levels of pluripotent hematopoietic stem cells rise up to 50-fold in the recovery phase after myelosuppressive chemotherapy and can be collected for autologous transplantation. In order to achieve circulating levels high enough to ensure a harvest capable of reconstituting a mature hematopoietic system after allogeneic donation, healthy donors must be “primed” with hematopoietic growth factors, using either rHuG-CSF or rHuGM-CSF. G-CSF is thought to stimulate HSC mobilization by decreasing SDF-1*α* gene expression and protein levels while increasing proteases that can cleave interactions between HSCs and the bone marrow environment [12]. Typical doses of G-CSF range between 2 and 24 *μ*g/kg administered daily to healthy donors [13], including donors above 60 years [14]. Mobilization and a subsequent increase in the concentrations of circulating HSCs are necessary to ensure adequate and successful collections.

The successful transplantation of hematopoietic stem/progenitor cells (HSPCs) is based on their ability to home to the BM niche and on their engraftment capacity. Interactions between HSPCs and their niches are altered during mobilization and must be reestablished during BM homing and repopulation. The homing of HSPCs to BM is a rapid process that takes place during the hours after transplantation and is an essential and necessary requirement for repopulation and engraftment [15].

Transplantation of hematopoietic progenitor cells (HPC) has become a widely accepted therapeutic option, particularly for patients with chemotherapy-sensitive hematological malignancies. Transplantation of HPC offers several advantages compared to bone marrow. Collection of HPC can be performed without general anesthesia, engraftment is faster, and supportive care and costs are reduced. HPC are harvested by leukapheresis after mobilization with chemotherapy and/or G-CSF. The use of mobilized peripheral blood is now the method of choice in autologous transplantation for various reasons, including an elevated production of immature cells, and, in comparison to the utilization of BM, the shorter time period required for a satisfactory repopulation, the more rapid engraftment, fewer technical difficulties, lower risk, and considerably less pain [16].

Although BM and peripheral blood are both still considered a source of stem/progenitor cells for this purpose [17, 18], peripheral blood is used in 71% of allogeneic transplantations [16].

A decisive factor for patients being transplanted in an autologous setting is the dose of transplanted HPC usually determined by measurement of CD34^+^ cells. Some data suggests that transplantation with less than 2 million of CD34^+^ cells/kg body weight (bw) is associated with a prolonged hematologic engraftment and worse outcome, whereas a dose of more than 5 million CD34^+^ cells/kg bw was of benefit [19]. In addition several data suggest that a minimum of 1.5 million [20], 2.5 million, or more than 5 million CD34^+^ cells might result in better outcome because of more rapid hematological engraftment and a decrease in infectious episodes [21]. Some data even suggest that patients might benefit from a dose higher than 15 million CD34^+^ cells/kg bw with regard to engraftment and hospitalization time after transplantation [22]. Therefore, 2–4 × 10^6^ CD34^+^ cells/kg body weight were recently defined as minimum and 8–10 × 10^6^ CD34^+^ cells/kg bw as optimum dose for autologous (tandem) transplantation in patients with multiple myeloma (MM) [23].

Number of publications state that the minimum level of CD34^+^ enumerated from the peripheral blood is around 14 cells/*μ*L [24], which in turn gives us an indication on the level of CD34^+^ cells that can be isolated after aphaeresis. Ideally around 3 million cells are collected per kg body weight. In our experiments, we have undertaken transplantations of individuals of different body weights. We feel that a universal figure like 14 cells/*μ*L is not adequate for transplantations for an 80–90 kg individual. Thus, multiple collections have to be undertaken from the same donor to get the required amount of 3 million cells/kg body weight. We feel that we need to establish uniformity in stem cell collection procedure from individuals of different kg body weights. Thus, our experiments are based on establishing a uniform factor that takes into account various factors such as the weight of the individual, CD34^+^ cells isolated/kg body weight of the individual, and the peripheral blood CD34^+^ enumeration.

## 2. Methods

### 2.1. Study Design

The study was approved by the local ethics committee for research. This study was part of the data of hematopoietic stem cell transplantation (HST) done at our institution. Transplantation involving peripheral blood stem cells is carried out on patients as part of the bone marrow transplantation procedure. Identity of patients is kept confidential and every sample in the laboratory is denoted by the laboratory identification number (Lab id). The data is taken from 20 subjects of different kg body weights to establish a uniform collection strategy.

### 2.2. Acceptance Criteria

The minimum sample that is accepted is 100 mL. A cut off CD34^+^ level of 14 cells/*μ*L is taken as a minimum level accepted but for this study we have also analyzed lower levels of CD34^+^ cells. Every patient is thoroughly screened for infectious diseases such as HIV and Hepatitis B and C, and the sterility tests done on every sample processed must be negative; as part of the stem cell donation program of our institution, every donor is subjected to other screening parameters (also called as the BMT workup) as recommended by the treating physician.

### 2.3. Introduction of Mobilizing Agents

We use cytokines such as filgrastim (glycosylated granulocyte colony-stimulating factor [G-CSF] which is a common mobilizing agent for haematopoiesis. This cytokine initiates the cascading sequence to produce committed mature blood cell components. Stem cell harvesting begins with enticing the CD34^+^ rich cell population out of the bone marrow niche environment as done earlier [25]. In order to achieve this, cytokines are exogenously administered to the patient in an effort to enhance the yield of cells within a short period of time.

### 2.4. Mobilization and Collection of Stem Cells

Success of a stem cell transplant depends on the mobilization of blood-forming CD34^+^ cells from the bone marrow. This level has to be analyzed so as to have an optimal level of CD34^+^ cells collected. We use an optimal value of 14 cells/*μ*L so as to ensure at least 2 × 10^6^ CD34^+^ cells/kg for a 70 kg individual. The stem cells were isolated at the patient's bedside by the Spectra Optia apheresis unit (Terumo BCT, USA) that is based on the COBE Spectra protocol where the mobilized cells are collected in a blood collection bag and excess plasma and RBC are sent back to the donor. The Spectra Optia is operated as per the manufacturer's guidelines and instructions for collection.

### 2.5. Processing and Cryopreservation of the Isolated Mononuclear Cells

After the apheresis procedure is completed in the hospital, the stem cell unit is brought to the laboratory for plasma reduction as part of the plasma reduction strategy. The concentration of the isolated CD34^+^ cells is calculated, thereby calculating the CD34^+^ cells isolated per kilogram body weight of the patient.

### 2.6. Enumeration of Stem Cells and Dose Calculation for Transplantation

The postprocessing analysis is done as a quality control analysis of the isolated stem cells. 1 mL of the postprocessing nucleated cell rich plasma is taken for the QC analysis. 500 *μ*L of this plasma is diluted to 5 mL with normal saline (1 : 10 dilution). 2.5 mL of this diluted plasma is taken for the hematology analysis, whereas the remaining 2.5 mL is taken for the flow cytometry analysis. The hematology analysis is done in the Hematology Section of our institution using the Sysmex XE 5000 (USA). We get the total nucleated cell count which is reported as the TNCC. Flow cytometry analysis is done using the FC 500 (Beckmann Coulter, USA) using the Stem Kit (Beckmann Coulter) for hematopoietic stem cell determination. This kit uses a combination of two antibodies such as CD34^+^-PE/CD 45^+^-FITC. In addition, viability of cells is determined by 7-AAD (7-Amino Actinomycin D). The viability dye is an analog of actinomycin-D that contains a substituted amino group at the 7th position of the chromophore. We analyze the total CD34^+^ cells from the total TNCC in the population as per the formula shown below.

(i) The formula is as follows:
  (a) CD34^+^ cells present/*μ*L of peripheral blood collected from the donor:
    (1) (Amount  of  Leucocytes  presentL×TDF × %CD34+  cells  enumerated)×(106×100)−1.
  (b) Total CD34^+^ cells to be Transplanted/kg body weight:
    (2) $$\begin{array}{c} \frac{\text{Total}\;\text{number}\;\text{of}\;\text{cells}/\text{L}\left(\text{Post}\right)\times \text{TDF}\times \%\text{CD}34^{+}\times \text{Vol}\;\text{in}\;\text{Lit}}{\text{Total}\;\text{Body}\;\text{weight}\;\text{of}\;\text{the}\;\text{patient}\times 100}, \end{array}$$

where TDF is total dilution factor = dilution factor of the leukocyte for hematological evaluation × difference in the dilution factor between hematological and flow cytometry evaluation.

## 3. Results

### 3.1. Comparative Analysis of the Number of Stem Cells Enumerated from the Peripheral Blood with the Body Weight of the Patient

**Figure 1:**
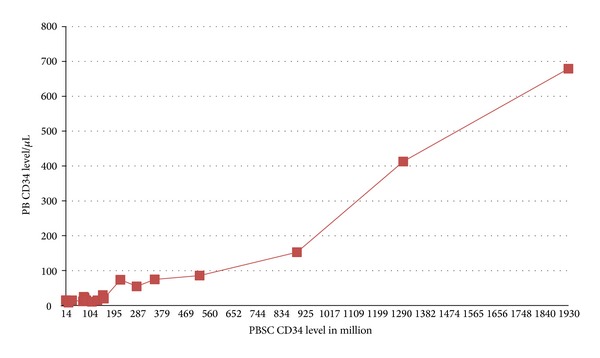
Graphical representation of the peripheral blood CD34^+^ level isolated from peripheral blood versus the final CD34^+^ level enumerated after processing.

A comparative analysis is carried out on three factors, namely, the number of CD34^+^ cells enumerated in the peripheral blood, the total CD34^+^ cells isolated per kg body weight of the patient, and the body weight of the patient (Table 1). All these three factors will determine a uniform formula that will be suitable for all collections so that a uniform amount of around 3 × 10^6^ cells are isolated at the end of each processing activity of stem cells. We have plotted a graph of the CD34^+^ level of the peripheral blood isolated per *μ*L versus the final CD34^+^ level isolated after the final collection (Figure 1).

**Figure 2:**
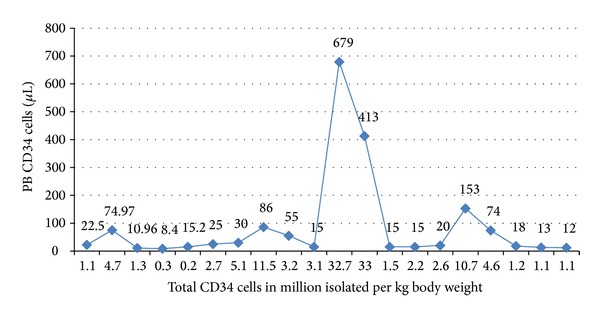
Graphical representation of the peripheral blood CD34^+^ level isolated from peripheral blood versus the CD34^+^ level enumerated/kg body weight.

We next plotted a graph that correlated the CD34^+^ level of the peripheral blood isolated per *μ*L versus the total CD34^+^ cells isolated per kg body weight (Figure 2).

## 4. Discussion

Successful bone marrow transplantation using hematopoietic stem/progenitor cells (HSPCs) is based on homing of HSPCs to BM and is a rapid process that takes place after transplantation. This process is called as engraftment which is the basis in haematopoiesis [15]. A decisive factor for patients being transplanted is the dose of transplanted HPC usually determined by measurement of CD34^+^ cells. Studies suggests that transplantation with less than 2 million of CD34^+^ cells/kg body weight (bw) is associated with a prolonged hematologic engraftment and worse outcome, whereas a dose of more than 5 million CD34^+^ cells/kg bw was of benefit [19]. It was recently seen that 2–4 × 10^6^ CD34^+^ cells/kg body weight were recently defined as minimum dose for transplantation in patients with multiple myeloma (MM) [23]. Thus, it is necessary to collect sufficient CD34^+^ cells in order to ensure a successful transplantation.

The aim of this study is to establish a suitable factor that can be multiplied by so as to get the desired CD34^+^ cells enumerated in the peripheral blood thus ensuring more than 3 × 10^6^ cells per collection irrespective of the weight of the patient. We have compared 4 factors namely, the CD34^+^ level isolated from peripheral blood, final CD34^+^ level isolated after each collection, CD34^+^ level per kilogram body weight of the patient, and the body weight of the patient (Table 1). We have noticed a definitive correlation between the number of CD34^+^ stem cells enumerated in the peripheral blood and the body weight of the patient. In the graphical representation (Figure 1), it is seen that a positive correlation exists between the peripheral blood CD34^+^ level and the total CD34^+^ level after each collection. To take this study further, we analyzed patients of different kilogram body weights with the CD34^+^ level collected from peripheral blood. When the CD34^+^ level in the peripheral blood was 15 cells/*μ*L, a sufficient number of CD34^+^ cells/kg body weight were isolated as long as the patient weighed under 20 kgs. For the kg body weight of 63 kgs, the peripheral blood CD34^+^ cells of 15 cells/*μ*L were grossly insufficient, making it mandatory to have multiple collections to get a desired limit of 2.5–5 × 10^6^ cells/kg body weight, thus putting a strain on the donor and prolonged stay in the hospital for cytokine administration such as G-CSF. A low CD34^+^ cells count in the peripheral blood such as 8 cells/*μ*L for an 87 kg person was highly inadequate. We noticed that maintaining the cutoff value (around 15 cells/*μ*L) for an 87 kg person was not a definitive approach.

**Figure 3:**
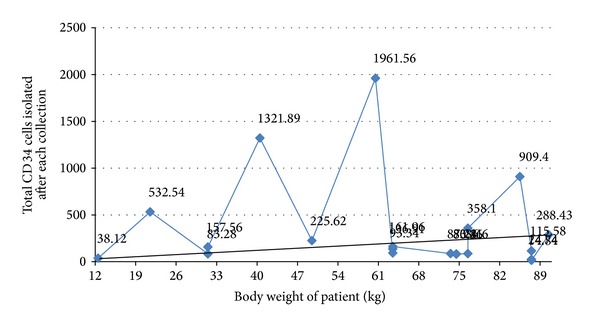
Graphical representation of the total CD34^+^ cells isolated after each collection procedure versus the body weight of the patient in kgs. A mean point is plotted from patients of different kg body weights but gives almost the same number of CD34^+^ progenitor cells enumerated per kg body weight.

We next plotted a graph of peripheral blood CD34^+^ cells enumerated from the peripheral blood versus CD34^+^ cells isolated per kg body weight (Figure 2). We found the presence of 4 peaks which represented higher than normal values for that patient. We now wanted to establish a relation between the total CD34^+^ cells isolated and the kg body weight of the patient (Figure 3). This graph again showed the presence of 4 prominent peaks indicating higher than normal CD34^+^ cells collected. We needed to normalize these values so as to get a suitable factor that can be used in transplantation. We maintained a requirement of 3 × 10^6^ cells for patients of different body weights across the spectrum, and we plotted a line joining the corresponding peripheral blood CD34^+^ levels (Figure 3). Thus, all points above and below this line are expected to yield higher or lower than the required 3 × 10^6^ cells, respectively. We next normalized this data to give the peripheral blood CD34^+^ cells required for 1 kilogram of body weight, and thus 25, 55, and 15 cells/*μ*L, respectively give 0.8, 0.6, 1.2 cells/*μ*L/kg, respectively. An average would be 0.8 cells/*μ*L/kg of the patient.

We thus have come up with a factor of 0.8 CD34^+^ cells/kg of the body weight that has to be considered before collection. The factor of 0.8 can be a suitable conversion factor to enumerate the minimum CD34^+^ cells required prior to transplantation. Thus, a 20 kg individual will need a baseline value of 20 × 0.8 = 16 CD34^+^ cells/*μ*L in the peripheral blood. As most literature have mentioned a cutoff value of around 14 cells/*μ*L [24], we have noticed that this number is sufficient for a 20 kg individual and more collections are required for an individual having a higher kg body weight. Thus, we are trying to create uniformity in the collection procedure based on the kilogram weight of the individual and the requirement for a single collection. More detailed investigation in this regard is required to establish a consistent result.

This paper assumes significant importance as, at the moment, the only accepted methodology in transplantation is minimal manipulation as per the standards. Expansion of HSCs does not come under the purview of an unmanipulated cell so we are forced to increase the efficiency of transplantation by limiting our strategy to an increased cell dosage as observed in the enumeration of the peripheral blood stem cells. We next plan to expand our study to include all the patients that are registered for stem cell transplantation.

Most of the stem cell transplants that have been carried out in our hospital are autologous cases. The patients are administered with premedication drugs such as ondansetron, dexamethasone, and lorazepam before the administration of cyclophosphamide (2000 mg/m^2^) followed by G-CSF (10 mcg/kg/day) until the day of apheresis.

## Figures and Tables

**Table 1:** Comparison study of the enumeration of stem cells isolated from peripheral blood from the donor, the body weight of the patient, and the total CD34^+^ cells enumerated.

Serial number	Peripheral blood CD34^+^ cells/*μ*L	Total CD34^+^ cells isolated	Body weight of the patient	CD34^+^ cells isolated/kg body weight
1	22.5	86.6 × 10^6^	76 kgs	1.1 × 10^6^
2	74.97	358.1 × 10^6^	76 kgs	4.7 × 10^6^
3	10.96	115.58 × 10^6^	87 kgs	1.3 × 10^6^
4	8.4	24.74 × 10^6^	87 kgs	0.3 × 10^6^
5	15.2	14.84 × 10^6^	87 kgs	0.2 × 10^6^
6	25	83.28 × 10^6^	31.2 kgs	2.7 × 10^6^
7	30	157.56 × 10^6^	31.2 kgs	5.1 × 10^6^
8	86	532.54 × 10^6^	21 kgs	11.5 × 10^6^
9	55	288.43 × 10^6^	90 kgs	3.2 × 10^6^
10	15	38.12 × 10^6^	12.3 kgs	3.1 × 10^6^
11	679	1961.56 × 10^6^	60 kgs	32.7 × 10^6^
12	413	1321.89 × 10^6^	40.1 kgs	33 × 10^6^
13	15	93.34 × 10^6^	63 kgs	1.5 × 10^6^
14	15	136.91 × 10^6^	63 kgs	2.2 × 10^6^
15	20	161.96 × 10^6^	63 kgs	2.6 × 10^6^
16	153	909.4 × 10^6^	85 kgs	10.7 × 10^6^
17	74	225.62 × 10^6^	49 kgs	4.6 × 10^6^
18	18	87.28 × 10^6^	73.5 kgs	1.2 × 10^6^
19	13	83.41 × 10^6^	74 kgs	1.1 × 10^6^
20	12	80.36 × 10^6^	74 kgs	1.1 × 10^6^
